# A trial-based economic evaluation of the Restore4Stroke self-management intervention compared to an education-based intervention for stroke patients and their partners

**DOI:** 10.1186/s12913-020-05103-x

**Published:** 2020-04-08

**Authors:** Ghislaine A. P. G. van Mastrigt, Mitchel van Eeden, Caroline M. van Heugten, Nienke Tielemans, Vera P. M. Schepers, Silvia M. A. A. Evers

**Affiliations:** 1 CAPHRI, School for Public Health and Primary Care, Department of Health Services Research, Faculty of Health, Medicine and Life Sciences, Maastricht University, P.O. Box 616, 6200 MD Maastricht, the Netherlands; 2 MHeNS, School for Mental Health and Neuroscience; Department of Psychiatry and Neuropsychology, Faculty of Health, Medicine and Life Sciences Maastricht University, P.O. Box 616, 6200 MD Maastricht, the Netherlands; 3 Department of Neuropsychology and Psychopharmacology, Faculty of Psychology and Neuroscience, Maastricht University, P.O. Box 616, 6200 MD Maastricht, the Netherlands; 4 Center of Excellence in Rehabilitation Medicine, Brain Center Rudolf Magnus, University Medical Center Utrecht, and De Hoogstraat Rehabilitation, Utrecht, The Netherlands; 5 Department of Public Mental Healthcare, Trimbos Institute, Netherlands Institute of Mental Health and Addiction, Postbus 725, 3500 AS Utrecht, The Netherlands

**Keywords:** Self-management, Stroke, Cost and cost analysis, Cost-effectiveness

## Abstract

**Background:**

Since stroke survivors are increasingly responsible for managing stroke-related changes in their own health and lifestyle, self-management skills are required. In a recent randomised controlled trial a self-management intervention based on proactive coping action planning (SMI) in comparison with an education-based intervention (EDU) in stroke patients was investigated. However, no relevant treatment effects on the Utrecht Proactive Coping Competence scale (UPCC) and the Utrecht Scale for Evaluation of Rehabilitation Participation (USER-Participation) were found. The current study is a trial-based economic evaluation from a societal perspective comparing the same interventions (SMI versus EDU).

**Methods:**

UPCC, USER-Participation and EuroQol (EQ-5D-3 L) and costs were measured at baseline, three, six and twelve months after treatment. For the cost-effectiveness analyses, incremental cost effectiveness ratios (ICERs) were calculated for UPCC and USER-Participation. For the cost-utility analyses the incremental cost utility ratio (ICUR) was expressed in cost per Quality Adjusted Life Years (QALYs). Outcomes were tested by means of AN(C)OVA analyses and costs differences by means of bootstrapping. Bootstrapping, sensitivity analyses and a subgroup analysis were performed to test the robustness of the findings.

**Results:**

One hundred thirteen stroke patients were included in this study. The mean differences in USER-Participation scores (95%CI:-13.08,-1.61, *p*-value = .013) were significant different between the two groups, this does not account for UPCC scores (95%CI:-.267, .113, p-value = not significant) and QALYs (p-value = not significant) at 12 months. The average total societal costs were not significantly different (95%CI:€-3380,€7099) for SMI (€17,333) in comparison with EDU (€15,520). Cost-effectiveness analyses showed a mean ICER of 26,514 for the UPCC and 346 for the USER-Participation. Cost-utility analysis resulted in an ICUR of €44,688 per QALY. Assuming a willingness to pay (WTP) threshold of €50,000 per QALY, the probability that SMI will be cost-effective is 52%. Sensitivity analyses and subgroup analysis showed the robustness of the results.

**Conclusions:**

SMI is probably not a cost-effective alternative in comparison with EDU. Based on the current results, the value of implementing SMI for a stroke population is debatable. We recommend further exploration of the potential cost-effectiveness of stroke-specific self-management interventions focusing on different underlying mechanisms and using different control treatments.

## Background

Stroke causes severe disability and may lead to long term chronic problems resulting in lifelong increased healthcare utilization. The growing demand for stroke care in combination with limited healthcare resources, has led, in the past few years, to an increased interest into the economic aspects of stroke [1]. The financial burden due to stroke are considerable as indicated by the following figures. In the European Union (EU) the total cost of stroke in 2015 was calculated as €45 billion. Forty four percent of this amount is related to direct health care costs e.g. in-hospital care (77%) [2]. Indirect medical costs like informal care costs were estimated at €15.9 billion (35%) and productivity losses €5.4 billion (12%) of stroke in the EU in 2015 [2].

Besides this economic impact, there is also a high disease burden related to stroke. One year after stroke, 35% of patients are functionally dependent, indicating that stroke is a leading cause of disability [3]. For both stroke survivors and their partners, stroke has long term consequences on their health related quality of life [4–6]. Since stroke survivors have become increasingly responsible for managing changes in their own health and lifestyle due to stroke, self-management skills are more and more required [7].

Currently, evidence regarding long-term stroke-specific self-management interventions is limited and no evidence is available on their cost-effectiveness. A recent systematic review into the cost-effectiveness of self-management interventions in chronic care found that the majority of these interventions were cost-effective in comparison with (mostly) care as usual alternatives, but these studies were generally subject to limited methodological quality [8]. In this review, no studies on the cost-effectiveness of stroke-specific self-management interventions could be identified. However, there is evidence regarding the effect on Quality of Life of stroke-specific self-management interventions [9]. These interventions focus mainly on the enhancement of cognitions underlying the intentions of behaviour (e.g. self-efficacy and control cognitions) in the short term, rather than teaching patients to anticipate the consequences of their stroke and develop corresponding solutions in advance [10]. In addition, teaching patients proactive coping strategies when confronted with a chronic disease have potential benefits [11, 12]. Patients who acquire self-management skills to cope with chronic consequences of their stroke may be less dependent on health care resources, as decreased healthcare utilization may lead to cost containment. Tielemans et al. have evaluated the effectiveness of the self-management intervention under investigation in the current economic evaluation and found little compelling evidence favouring the self-management intervention based on proactive coping planning (SMI) over the a stroke-specific education-based intervention (EDU) [13]. Based on the current evidence, this study aims to evaluate the cost effectiveness of SMI in comparison with EDU from a societal perspective in stroke patients [14]. The results of study will help policymakers to decide whether to implement the SMI intervention considering both effectiveness as well as cost-effectiveness.

## Methods

### Aim

To evaluate the cost-effectiveness and cost-utility of a stroke-specific self-management intervention based on proactive coping action planning in comparison with a stroke-specific education-based intervention from a societal perspective.

### Design

The current study describes an economic evaluation attached to the Restore4Stroke Self-Management Study: a multi-centre randomized controlled trial (RCT) with a two-group parallel design, using a balanced randomization stratified by the participating hospital or rehabilitation centre (1:1 ratio). The Medical Ethics Committee of the University Medical Center Utrecht and the ethics committees of the participating institutes approved this study. Of all stroke patients and their partners written consent was obtained. The RCT was registered in the Dutch Trial Register as NTR3051 [15]. The current study reports only patient data. Detailed information on the study protocol can be found elsewhere [1, 16]. For this study, general methods for performing and reporting economic evaluations in health care were applied [17, 18].

### Setting of the study

The study was conducted between February 2012 and May 2014 at the outpatient facilities of three hospitals (two general and one university) and five rehabilitation centres spread across the Netherlands.

### The characteristics of participants

Stroke patients (≥ 18 years) who suffered a first or recurrent symptomatic stroke (i.e. ischemic or intracerebral haemorrhagic) at least six weeks prior to recruitment, confirmed by a neurologist, were eligible for this study. Furthermore, participation problems experienced by the patients according to the restriction scale of the Utrecht Scale for Evaluation of Rehabilitation Participation (USER-Participation) were used to select eligible patients [19].

Patients were excluded if they were clinically judged as having insufficient mental abilities to understand and benefit from the intervention, or that the production or comprehension of language would be disturbed (score below 5 on the shortened version of the Aphasia Scale of the Dutch Aphasia Foundation, SAN [20]), behavioural problems hampering functioning in a group, or if there were major depression, or if they were already receiving structured psychological counselling aimed at proactive coping strategies post stroke at the time of recruitment.

### Intervention and comparator

#### Self-management intervention (SMI) for stroke

SMI lasted ten weeks. In the first six weeks, weekly two-hour sessions took place and patients had one two-hour booster session in the tenth week. The settings were outpatient facilities of hospitals and rehabilitation centres in the Netherlands. A group-based treatment was given at four to eight participants (with a maximum of four stroke patients and their partners, if applicable) by two rehabilitation medicine professionals (i.e. a psychologist and a social worker). The trainers received a one-day training on content SMI and the importance of adhering to the treatment protocol. The SMI consisted of three parts; a) Teaching patients and partners in proactive action planning strategies embedded in four themes: ‘handling negative emotions’, ‘social relations and support’, ‘participation in society’, and ‘less visible consequences of stroke’. B) Educate about; “stroke consequences” and same themes mentioned in part A. and C) Peer support.

#### Control treatment (EDU)

EDU lasted ten weeks, with three one-hour sessions in the first six weeks and one one-hour booster sessions in the tenth week. The setting and the number of participants per group were the same as for SMI. The treatment was provided by one rehabilitation medicine professional (i.e. a psychologist or a social worker). Training of providers consisted of a one and half hours training on the content of the EDU and the importance of adhering to the treatment protocol. In the control treatment the following three themes were discussed: ‘the brain and stroke’, ‘general consequences of stroke’, and ‘preventing a recurrent stroke’ themes.

#### Justification of choice for the control intervention

Choosing EDU as a control intervention allowed us to actively control this group, to compare the self-management intervention with group therapy and a form of education, and to aim on the potential effectiveness of the proactive coping element of SMI.

#### Procedure

Rehabilitation physicians and nurse practitioners selected eligible stroke patients through finding cases at outpatient facilities. Qualifying patients who had their regular consultation at the outpatient facility of the participating hospital or rehabilitation centre were invited to participate in the study. Patients were given an information leaflet if they showed interest in participating in the study. After five days, the primary researcher (NT) called patients and partners to find out if they wanted to participate. When a group of eight patients was composed, the primary researcher/research assistant conducted baseline measurements at the patient’s home or at the participating centre. At the end of the baseline assessment, patients were randomized into either SMI or EDU.

Patients and partners were informed about the comparison of two education-based interventions. The randomization took place via a closed envelope opened by the patient; hence the outcome researchers and research assistants were blind to the allocated intervention at baseline wherever possible. All questionnaires were filled out by the participants, with or without help from a research assistant. They could be completed either digitally or on paper, and if necessary an appointment with a research assistant could be made to assist with the assessment.

#### Time horizon

After baseline assessment (T0), patients and partners were randomly allocated to one of the treatment options. Follow-up assessments took place immediately after finishing the intervention, approximately three months post baseline (T1), at six months post baseline (T2) and twelve months post baseline (T3). A time frame of 12 months was chosen as this is expected to be long enough to adequately evaluate the cost-effectiveness of this intervention.

#### Outcome measures

The outcomes for the cost-effectiveness analyses (CEA) were proactive coping and participation. Proactive coping was assessed with the Utrecht Proactive Coping Scale (UPCC), which is a 21-item self-assessment tool scored on a 4-point scale ranging from ‘not competent at all’ to ‘competent’. A total score was computed by averaging all item scores (range 1–4), where higher scores indicate higher levels of proactive coping. The psychometric properties of this scale have proven to be good for stroke patients and healthy elderly people (mean age 62.3 years (SD 5.4)) [11, 12]. Participation was assessed with the Utrecht Scale for Evaluation of Rehabilitation Participation (USER-Participation), an eleven-item self-assessment tool scored on a 4-point scale ranging from ‘not possible at all’ to ‘independent without difficulty’. A total score was calculated by adding up all items and transforming the resulting sum score into a score on a 0–100 scale, where higher scores indicated lower levels of restriction of participation hence better participation. Participation is a domain of the International Classification of Functioning, Disability and Health. The psychometric properties of this scale have proven to be satisfactory for rehabilitation outpatients, including stroke patients [19, 21, 22].

The outcome for the cost-utility analysis (CUA) was Quality Adjusted Life Years. For this the health related quality of life was measured by means of the EuroQol descriptive system containing three levels (EQ-5D-3 L). It consists of five items measuring the following five dimensions: mobility, self-care, usual activities, pain/discomfort and anxiety/depression. The Dutch tariff was used to estimate the utility of health states described by patients [23]. Quality adjusted life years (QALYs) were calculated by means of the area under the curve method.

#### Estimating resource use and costs

Cost data were collected from a societal perspective through a specially designed 19-item self-reported cost-questionnaire. The feasibility and validity of generic self-reported instruments has been investigated previously [24]. Two main cost categories were distinct: healthcare costs and non-healthcare costs (intervention-, patient- and family-, and productivity costs).

Intervention costs for each intervention were calculated by means of a bottom-up approach. For the SMI, these included the costs of hourly wages of the medical professionals involved, day training, if necessary, for these professionals, workbook for professionals and workbook for patients. For EDU, hourly wages of the medical professionals involved, 1.5 h education training, if necessary, for these professionals, workbook for professionals and workbook for patients were taken into account. The Dutch Manual for Costing [25] was used as a guideline for calculating healthcare costs except for medication costs. Healthcare costs covered health care utilization (e.g. general practitioner (GP) and medical specialist consultations), alternative care, medication and home care. The costs of prescription drugs were valued based on the price per dosage for drug costs in the Netherlands [26, 27], and medical and personal aids were calculated per user within the aid category provided by the Dutch Care institute (www.gipdatabank.nl). Patient- and family costs included the cost of informal care and travel costs. Informal care was valued as a ‘shadow price’, meaning the hourly wage rate of a professional caregiver (i.e. housekeeper). Travel costs were calculated by multiplying the average distance with standard price weights provided by the Dutch Manual for Costing [25], which corrected for public transport costs and parking costs. Productivity costs were valued according to the human capital approach [28]. This approach states that productivity costs are calculated by multiplying the number of sick days by labour costs, corrected for different age categories.

#### Currency, price date and conversion

All costs reported are expressed in Euro’s (€). The year 2012 was used as index year Indexing was done using price induces [29]. Discounting was not applied in this economic evaluation, since the follow-up period did not exceed one year.

#### Analytical methods

All statistical analyses were performed using the Statistical Package for the Social Sciences (SPSS) version 21. All data were analysed according to the intention-to-treat principle. Missing data were handled by individual mean imputation, the recommended imputation method for dealing with intermittent data in economic evaluation studies [30]. The potential impact of the imputation method used was investigated by comparing baseline characteristics of patients who did not had any missings on the study outcomes (completers) and those who had one of the outcomes missing. We also studied the impact of imputation on the study outcomes by means of subgroups analysis (see section sensitivity and subgroup analysis for more details).

The baseline characteristics (e.g. demographic, stroke related and costs) between SMI and EDU patient groups were analysed using non-parametric Mann-Whitney test or Chi-Square tests. Baseline differences of UPCC, USER-Participation, utilities and differences in QALYs between both groups were tested using ANOVA. Since baseline utility measurements are included when calculating QALYs we consider any baseline difference in utility scores as a potential bias, regardless of whether this difference is significant or not. Therefore, we used a regression based correction method to correct for baseline differences in utility scores [31]. To investigate differences between the two groups at 3, 6 and 12 months follow up analyses of covariance (ANCOVA) were performed for UPCC, USER-Participation and utilities using baseline scores as covariates.

We calculated an incremental cost-effectiveness ratio (ICER) by dividing the incremental costs by the incremental effects. The incremental cost-utility ratio (ICUR) was estimated by dividing the incremental costs by the differences in QALYs. Because cost data is generally known to be skewed, we used non-parametric bootstrapping to estimate the uncertainty surrounding the cost-effectiveness ratio. Different sets of replication runs were tested, and 5000 replications results in stable outcomes. Cost-effectiveness planes (CE-planes) were drawn by presenting the bootstrapped cost-effectiveness and cost-utility pairs. Statistically significant differences in costs were determined by means of a 95% confidence interval (95%CI). If the CI entailed a ‘0’ value, no statistical differences in costs were found. The probability of the self-management intervention being a cost-effective alternative in comparison with the education-based intervention is demonstrated by a cost-effectiveness acceptability curve (CEAC). A CEAC shows the probability of an intervention being a cost-effective alternative for a certain threshold; the amount of money society is willing to pay (WTP) to gain one unit of effect. Both CEAs present costs per one-point improvement on the USER-Participation and UPCC, and the CUA presents costs per one QALY gained. The WTP threshold for the UPCC and USER-Participation is an unknown quantity, and the WTP for a QALY differs per country. In the Netherlands, the Care Institute advised to use depending on the disease severity different thresholds. More, specific €20.000 for mild, €50.000 moderate and €80.000/QALY for severe burden of disease. We categorised the population of this study as having a moderate disease burden and therefore we used a threshold of €50,000 [32].

#### Sensitivity analyses and subgroup analysis

We performed five one-way sensitivity analyses and one subgroup analysis. First, the unit costs of rehabilitation day treatment were decreased to €116.81, equal to a regular rehabilitation contact. Second, the friction cost method was used instead of the human capital approach to estimate productivity costs [25]. Third total societal costs versus total healthcare costs were analysed because choice of perspective is an ambivalent subject [33]. Fourth, to investigate the potential impact of using a specific baseline difference correction method, we used the Delta QALY method instead of the regression correction used in the base case analyses. Fith, as different sets of tariffs exist for calculating utilities, we analysed the impact of using Dutch tariffs versus UK tariffs [34]. Finally, we performed one subgroup analysis using the complete cases to investigate the impact of the imputation method on the study conclusions. All sensitivity analyses were predefined and the subgroup analysis was done on recommendation of the statistical reviewer.

## Results

### Sample

**Fig. 1:**
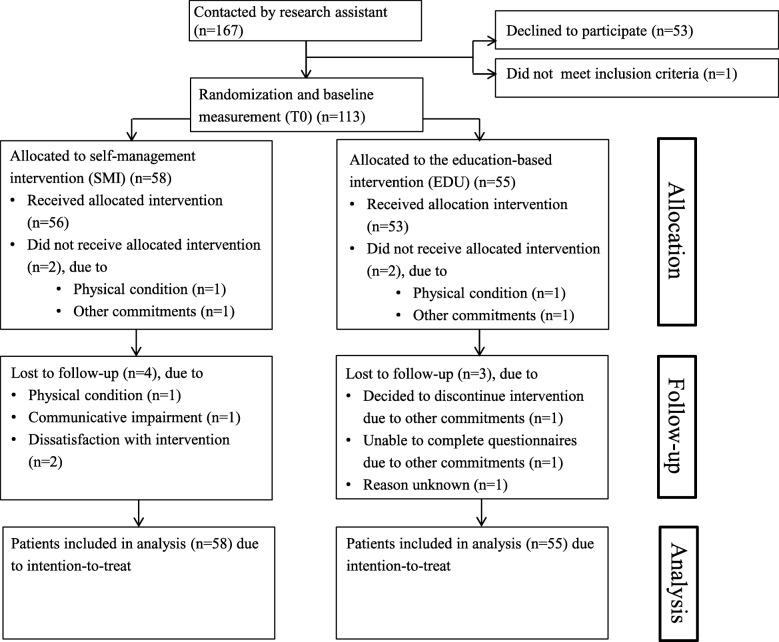
Inclusion of patients

Between February 2012 and May 2013, 167 post stroke patients from three hospitals and five rehabilitation centres in the Netherlands were contacted by NT (Fig. 1). One patient did not meet the inclusion criteria and was excluded, and 53 patients declined to participate. One hundred thirteen patients were eligible for inclusion. Fifty-eight (51%) patients were allocated to the self-management intervention and fifty-five (49%) patients to the education intervention. Eight patients were lost during follow-up hence one hundred five (93%) patients completed the interventions. Since all analyses were based on the intention-to-treat principle, one hundred thirteen patients were included. In the SMI group less males were included and also three stroke related characteristics were somewhat different from EDU group (Post stroke in months, Type of stroke and Stroke history). Further details of baseline characteristics can be found in Table 1.

**Table 1:** Patients’ characteristics at baseline (*n* = 113)

	SMI (*n* = 58)		EDU (*n* = 55)		*p*-value
	n		n		
*Demographic characteristics*					
Mean age in years (SD)	58	55.2 (8.9)	55	58.8 (8.7)	.056
Gender (% man)	58	44.8	55	60.0	.038^a^
Educational level (% low)	56	69.6	54	63.0	.115
Living with partner (%)	57	73.1	55	76.9	.254
Employment status (% employed)	58	22.4	55	23.6	.675
Ethnicity (% Dutch nationality)	58	98.3	54	100	.344
*Stroke characteristics*					
Post stroke in months (SD)	54	15.6 (20.9)	55	21.9 (34.1)	.041^a^
Type of stroke (% infarction)	55	78.2	55	87.3	.029^a^
Affected hemisphere (% right)	54	44.4	55	45.5	.870
Stroke history (% recurrent)	54	13.0	55	21.8	.039^a^
*Costs*					
Healthcare costs, € (SD)	58	2113.4 (413.4)	55	2390.4 (407.5)	.179
Non-healthcare costs, € (SD)	58	4259.2 (671.3)	55	3269.7 (545.5)	.052
Total societal costs, € (SD)	58	6387.4 (827.9)	55	5648.8 (761.0)	.093

Non-parametric Mann-Whitney test or Chi-Square test used to test for significant differences between SMI and EDU

*SD* standard deviation

^a^significant at the 0.05 level

### Missings

For utilities fourteen patients (12.4%), UPCC twelve patient (10.6%) and USER-Participation twelve patients (10.6%) had missing questionnaires. For the costs questionnaires this was somewhat higher but only twenty one patients had more than three items missing on the four measurement moments. We compared completers and non-completers at several baseline characteristics (age, gender, living with partner, type of stroke, baseline scores of primary outcomes and costs) for these two groups and could not detect any differences between the completers and non-completers.

### Cost analysis

The average total societal costs were not significantly different for SMI (€17,333) in comparison with EDU (€15,520), with a 95%CI of €-3380, €7099. This also accounts for the average total healthcare cost (€6138 compared to €5899) and total non-healthcare costs (€11,320 compared to €9507), with 95%CI of €-2352, €2685 and €-1923, €5769 respectively. Three cost categories show a significant difference between both groups; activity therapy is significantly higher for SMI (€276) in comparison with EDU (€65) (95%CI €24, €452); tools, and home adjustments were both significantly higher for EDU (€231 and €192 respectively) (95%CI (€-272, €-3)) in comparison with SMI (€106 and €22) (95%CI of (€-359, €-16)). Although not significantly different, productivity costs were much larger for SMI (€5392) in comparison with EDU (€4187). Intervention costs were larger for SMI (€764 in comparison with €222 for EDU). Further details are presented in Table 2.

**Table 2:** Average resource use and costs (Euro’s) per category over 12 months (bootstrapped)

Category	Unit price	SMI (***n*** = 58)		EDU (***n*** = 55)		95%CI ^a^
		Average use (SD)	Average costs, mean (median, min, max)	Average use (SD)	Average costs, mean (median, min, max)	
*Healthcare*						
Hospital	Night	1.0 (2.4)	491.8 (483, 0, 5338)	1.5 (4.1)	698.0 (676, 0, 8492)	(− 881, 356)
Rehabilitation centre	Night	0.0 (0.0)	0.0 (0, 0, 0)	0.1 (0.3)	13.1 (13, 0, 722)	(−39, 0)
Nursing home	Night	0.0 (0.0)	0.0 (0, 0, 0)	0.8 (6.1)	209.3 (207, 0, 11,373)	(−620, 0)
General practitioner	Contact	13.3 (17.0)	398.5 (391, 0, 2812)	11.0 (11.0)	331.7 (329, 0, 1715)	(−77, 228)
Specialist	Contact	9.9 (14.4)	1250.7 (1237, 0, 10,637)	8.0 (8.8)	1001.2 (994, 0, 5698))	(− 236, 805)
Physiotherapy	Contact	22.4 (33.9)	855.4 (857, 0, 5964)	29.5 (40.6)	1132.2 (1122, 0, 5964)	(− 803, 248)
Remedial therapy	Contact	8,1 (18.6)	303.4 (297, 0, 3178)	10.3 (25.2)	373.2 (369, 0, 4516)	(− 363, 227)
Mensendieck	Contact	0.0 (0.0)	0.0 (0, 0, 0)	0.1 (0.6)	2.9 (3, 0, 167)	(−9, 0)
Occupational therapy	Contact	0.7 (3.2)	15.5 (14, 0, 444)	0.9 (3.7)	20.0 (20, 0, 467)	(−34, 24)
Activity therapy	Contact	7.3 (23.5)	275.6 (268, 0, 4349)	1.7 (5.0)	64.6 (62, 0, 966)	(24, 452)^b^
Speech therapy	Contact	1.6 (4.9)	53.8 (52, 0, 753)	2.8 (12.6)	100.1 (93, 0, 3119)	(−192, 54)
Social work	Contact	2.2 (4.9)	146.0 (143, 0, 1657)	2.2 (5.2)	155.7 (153, 0, 2071)	(−137, 109)
Psychologist	Contact	2.6 (5.4)	216.1 (214, 0, 2001)	4.8 (12.7)	401.2 (391, 0, 6058)	(− 504, 84)
Psychiatric nurse	Contact	0.1 (0.6)	3.2 (3, 0, 91)	0.1 (0.6)	3.4 (3, 0, 91)	(−7, 6)
Psychiatrist	Contact	0.4 (1.3)	42.1 (41, 0, 656)	0.2 (1.0)	24.9 (24, 0, 656)	(−31, 64)
Rehabilitation day treatment	Day	7.1 (17.8)	1883.3 (1831, 0, 24,521)	4.2 (13.7)	1123.9 (1066, 0, 22,522)	(− 809, 2271)
Medication	Other		190.1 (187, 0, 984)		289,1 (288, 0, 1454)	(− 202, 6)
**Subtotal (SD)**			**6138.2 (832.1)**		**5898.8 (986.1)**	**(−2352, 2685)**
*Non-healthcare*						
Travel costs	Other		342.9 (343, 0, 1202)		336.8 (335, 6, 1762)	(−132, 132)
Productivity costs	Hours/week	5.6 (9.6)	5392.4 (5322, 0, 39,107)	4.4 (7.7)	4187.3 (4144, 0, 29,997)	(− 1943, 4421)
Productivity costs partner	Hours/week	1.0 (2.4)	955.9 (929, 0, 10,546)	1.4 (4.3)	1360.6 (1319, 0, 24,373)	(− 1704, 784)
Paid help	Hours	49.7 (108.9)	1837.6 (1794, 0, 20,518)	50.1 (140.6)	1838.9 (1762, 0, 32,858)	(− 1692, 1564)
Unpaid help	Hours	141.7 (256.1)	1885.9 (1862, 0, 17,171)	88.3 (158.6)	1170.0 (1168, 0, 12,009)	(− 244, 1814)
Tools^c^	Item	0.6 (1.1)	105.7 (104, 0, 1077)	0.9 (1.2)	230.9 (226, 0, 2286)	(−272, −3)^b^
Home adjustments^c^	Item	0.2 (0.6)	22.4 (22, 0, 384)	0.3 (0.6)	192.0 (187, 0, 3843)	(−359, −16)^b^
**Subtotal (SD)**			**11,320.2 (1469.8)**		**9506.9 (1360.1)**	**(−1923, 5769)**
*Intervention costs*	Other		764.2		222.1	
**Total societal costs (SD)**			**17,333.2 (1813.7)**		**15,520.1 (2028.9)**	**(−3380, 7099)**

^a^95% Confidence Interval level

^b^significant difference

^c^Tools: e.g. brace, special glasses; Home adjustments: e.g. toilet or shower adjustment

### Outcomes

At baseline, three and six months follow up, the mean differences of USER-Participation, UPCC and utilities are not significantly different between the two groups. At 12 months the mean UPCC scores (95%CI -13.08,-1.61, *p*-value = .013) are statistically significant higher in the SMI compared to EDU group. For the other outcomes at 12 months, no differences between the groups were found, for details, check Table 3.

**Table 3:** Data of the three outcomes for SMI (*n* = 58) and EDU (*n* = 55) patients

	Baseline		ANOVA	three months		ANCOVA*		Six months		ANCOVA*	95% CI	12 months		ANCOVA*	
	SMI	EDU		SMI	EDU	Group		SMI	EDU	Group		SMI	EDU	Group	
	Mean (SD)	Mean (SD)		Mean (SD)	Mean (SD)	p-value	95% CI	Mean (SD)	Mean (SD)	p-value		Mean (SD)	Mean (SD)	p-value	95% CI
**UPCC**	2.9 (.57)	2.9 (.51)	.945	2.9 (.58)	2.9 (.58)	.728	−0.22, 0.15	3.0 (.62)	2.9 (.55)	.233	−0.31, 0.08	2.94 (0.69)	2.87 (0.58)	.421	− 0.267,0.113
**USER-Participation**	70.9 (15.5)	73.4 (16.6)	.423	70.9 (15.1)	71.5 (18.8)	.689	−6.97,4.62	70.3 (16.2)	70.5 (18.4)	.645	−6.95,4.33	73.1 (16.6)	67.6 (19.6)	.013**	−13.08,-1.61
EQ-5D-3 L															
Utility	.60 (.31)	.68 (.25)	.158	0.71 (.21)	0.69 (.19)	.051	−0.12, 0.00	0.71 (.21)	.70 (.22)	.330	−0.10,0.04	0.72 (.24)	.68 (.24)	.099	−0.15,0.013
												**12 months**		**ANOVA**	
												**SMI**	**EDU**		
QALY***												0.715 (.140)	.672 (.118)	.080	

*SMI* Self-management intervention, *EDU* education based intervention, *UPCC* Utrecht Proactive Coping Scale, *USER-Participation* the Utrecht Scale for Evaluation of Rehabilitation Participation, *EQ-5D-3 L* EuroQol descriptive system with three levels, *QALY* Quality Adjusted Life Years, *ANCOVA* Analysis of covariance, *SD* standard deviation, *95 CI%* 95% confidence interval

*with baseline score as covariate

**significant *p*-value < 0.05

***Corrected QALY for baseline utility differences

### Cost-effectiveness and cost-utility analyses

**Fig. 2:**
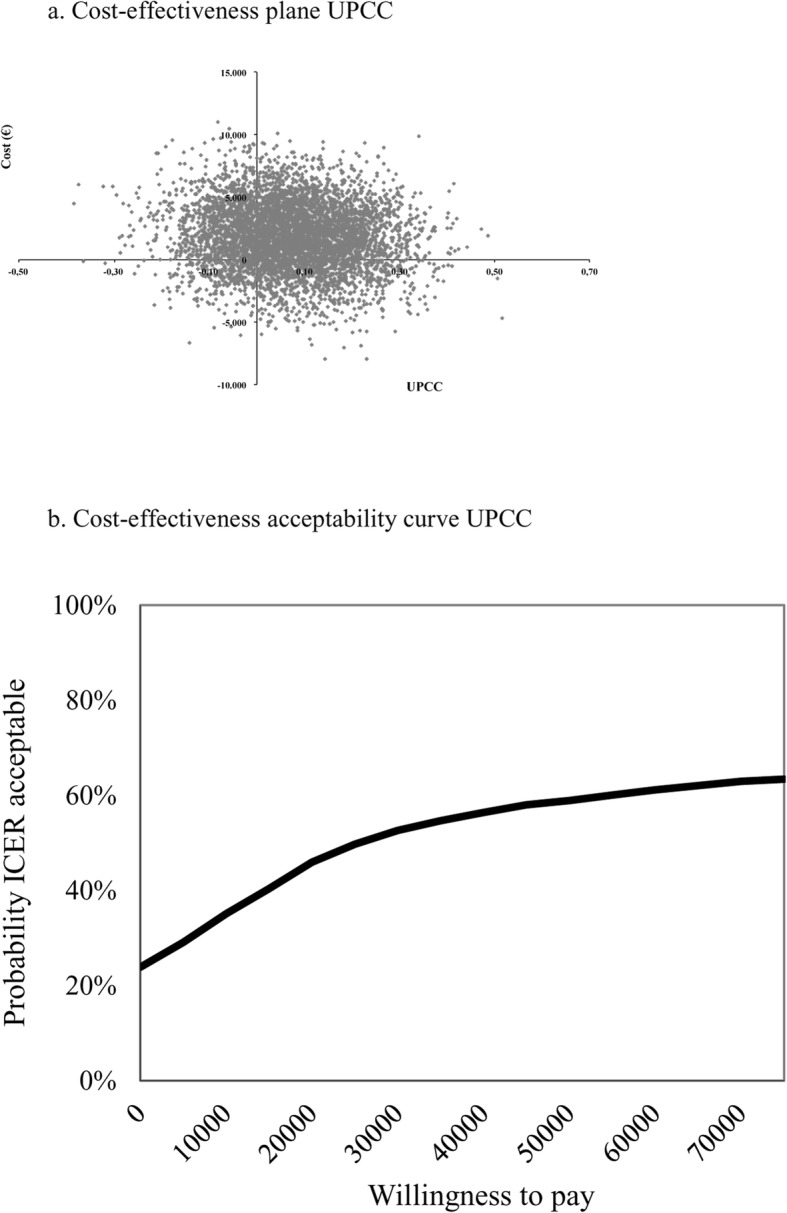
**a** Cost-effectiveness plane UPCC and **b** cost-effectiveness acceptability curve UPCC

**Fig. 3:**
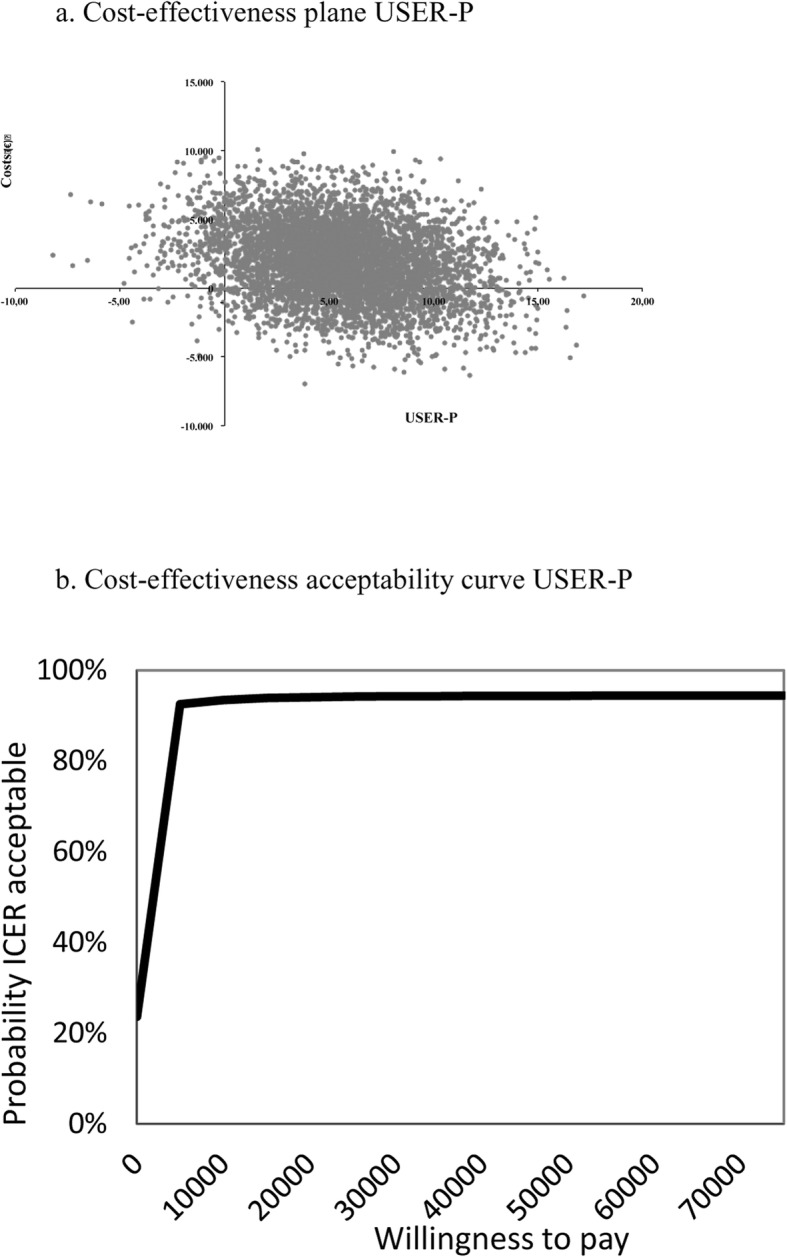
**a** Cost-effectiveness plane USER-Participation and **b** cost-effectiveness acceptability curve USER-Participation

**Fig. 4:**
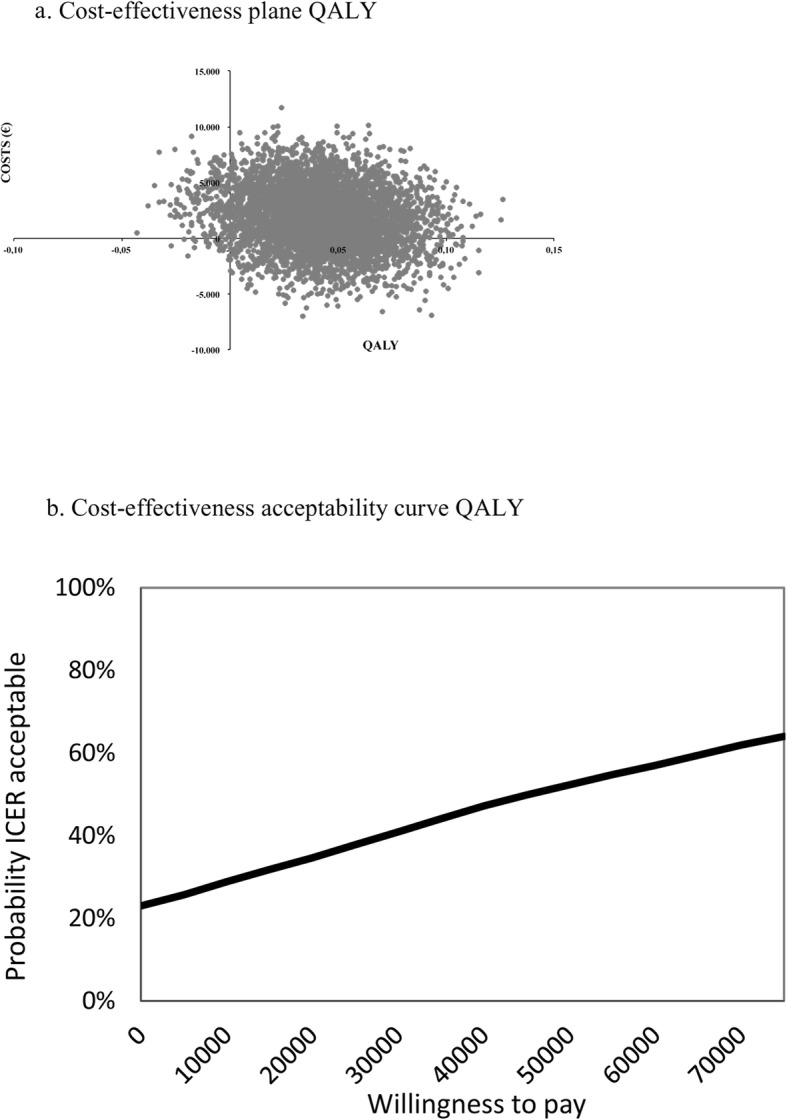
**a** Cost-effectiveness plane QALY and **b** cost-effectiveness acceptability curve QALY

Table 4 shows a minimal difference in effect on UPCC (.07 in favour of SMI) and higher costs for SMI (€1922) resulting in an ICER of €26,514. The majority of bootstrapped ICERs (54%) were located in the northeast (NE) quadrant of the CE plane (Fig. 2) indicating higher effects and higher costs; 22% of the bootstrapped ICERs are located in the dominant southeast (SE) quadrant, indicating higher effects and lower costs. The higher effects on USER-Participation for SMI (5.56) resulted in an ICER of €346. As also shown in Fig. 3, the majority of bootstrapped pairs (72%) were again located in the NE quadrant (higher effects and higher costs) of the CE plane while 23% were located in the SE quadrant (higher effects and lower costs). This slightly higher QALY for SMI, resulted in an ICUR of €44,688. Figure 4 shows that 73% of the bootstrapped pairs were located in the NE quadrant (higher effects and higher costs) of the CE-plane and 23% are in the SE quadrant (higher effects and lower costs).

**Table 4:** Bootstrapped results of the base case, sensitivity and subgroup analyses of all three study outcomes (UPCC, USER-Participation and QALYs)

Analysis^**a**^	Patients		ΔCosts (€)	ΔEffects	ICER	Distribution cost-effectiveness plane (quadrant,%)^**b**^			
	SMI	EDU				NE	SE (dominant)	SW	NW (inferior)
**Base case UPCC**	**58**	**55**	**1921.6**	**0.07**	**26,514.3**	**54**	**22**	**6**	**18**
**Sensitivity analyses**	58	55							
Unit price rehabilitation day			1489.5	0.07	20,511.8	52	21	5	22
Friction costs			784.3	0.07	10,821.2	47	27	8	18
Healthcare perspective			164.0	0.07	2263.3	41	33	11	15
**Subgroup analysis**	48	44							
Complete cases			3143.0	0.12	26,167.1	69	13	1	17
**Base case USER-Participation**	**58**	**55**	**1921.6**	**5.56**	**345.6**	**72**	**23**	**0**	**5**
**Sensitivity analyses**	58	55							
Unit price rehabilitation day			1489.5	5.56	267.9	68	27	1	4
Friction costs			784.3	5,56	141.1	60	35	1	4
Healthcare perspective			164.0	5.56	29.5	53	42	1	4
**Subgroup analysis**	48	44							
Complete cases			3143.0	4.86	646.3	77	14	0	9
**Base case QALY**	**58**	**55**	**1921.6**	**0.04**	**44,687.9**	**73**	**23**	**0**	**4**
**Sensitivity analyses**	58	55							
Unit price rehabilitation day			1489.5	0.04	34,638.6	70	26	1	3
Friction costs			784.3	0.04	18.238.4	61	36	1	2
Healthcare perspective			164.0	0.04	3814.6	53	43	1	3
Delta QALY			1921.6	0.11	17,970.3	75	24	0	1
QALY UK tariff			1921.6	0.05	39,744.8	75	23	0	2
**Subgroup analysis**	48	44							
Complete cases			3143.0	0.05	57,702.2	85	14	0	2

*ICER* incremental cost-effectiveness ratio, *SMI* self-management intervention, *EDU* education-based intervention

^a^Base case analysis values a rehabilitation day as a hospital treatment day (€266.53), calculates production costs by means of human capital method, uses the societal perspective to calculate total costs, corrects for baseline differences with regression analysis and calculates utilities with a Dutch tariff

Sensitivity analyses values a rehabilitation treatment day as a rehabilitation contact (€116.81), calculates production costs with the friction cost method, estimates total cost from a healthcare perspective, correction for baseline differences in utility scores with Delta QALY, utilities are calculated with a UK tariff, Subgroup analysis values complete cases defined as patients with more than 3 items missing on primary study outcomes (*n* = 44 education-based intervention and *n* = 48 self-management intervention

^b^NE (northeast quadrant): SM more effective and more costly compared to Edu

SE (southeast quadrant): SM more effective and less costly compared to Edu

SW (southwest quadrant): SM less effective and less costly compared to Edu

NW (northwest quadrant): SM less effective and more costly compared to Edu

The UPCC and USER-Participation CEACs are presented in Fig. 2 and Fig. 3. The slope of the UPCC CEAC indicates that with a WTP threshold of €37,500, the probability of SMI being cost-effective is 56%. This is in contrast with the USER-Participation slope, where a minimum WTP threshold of €2500 results in a 90% probability that SMI will be cost-effective. If a threshold of €50,000 is applied for cost/QALY [32], there is a 52% chance that SMI will be cost-effective (Fig. 4).

### Sensitivity analyses and subgroup analysis

The results of the one-way sensitivity analysis estimating a lower unit price for rehabilitation day treatment showed robustness of results for all three outcome measures. Similar to the base case analyses, between 21 and 27% of the bootstrapped ICERs/ICURs were located in the SE quadrant and a slightly lower percentage of bootstrapped ICERs were located in the NE quadrant. Using the friction cost method instead of human capital approach for estimation of the productivity costs resulted in a slight increase in the percentage of bootstrapped ICERs/ICURs located in the SE quadrant and a slight decrease in the NE quadrant for all three-outcome measures. An increase of 5% of bootstrapped ICERs in the SE quadrant was found for the UPCC, and an increase of 12% of bootstrapped ICERs/ICURs for both the USER-Participation and QALY. Only taking into account the healthcare costs resulted in lower ICERs/ICURs for all three study outcomes since fewer cost categories were included. Accordingly, this resulted in a shift in the distribution of bootstrapped ICERs/ICURs in favour of using a healthcare perspective since the percentage in the NE quadrant decreased, while increasing in the SE quadrant. One-third of bootstrapped ICERs were located in the SE quadrant for the UPCC (33%) and 42% ICERs and 43% ICURs for the USER-Participation and QALY respectively. Using a Delta QALY correction method to estimate HRQoL and UK tariffs to calculate QALYs resulted in largely similar distribution of ICURs in comparison with the base case analysis. The complete cases analyses shows that there is shift of ICERs/ICURs from the SE quadrant to the NE quadrant. At a WTP threshold of €50.000 for the cost/QALY there is a 45% change of ICUR acceptance in the subgroup analyses.

Further details on the sensitivity analyses and subgroup analysis are shown in Table 4.

## Discussion

To our knowledge, this the first full economic evaluations of self-management intervention for post stroke patients. Although the importance was recognised [9, 35] and ongoing studies are identified [36–39], no economic evaluation relating to this topic was published yet. The USER-Participation scores were significant different between the two groups, this does not account for UPCC, Utilities and QALYs. The results also indicate a stroke-specific self-management intervention based on proactive coping action planning (SMI) is more expensive than a stroke-specific education-based intervention. The higher costs for self-management intervention were mainly explained by higher productivity costs and significantly higher costs for tools, home adjustments and activity therapy. Also, intervention costs were higher for SMI which is evident because SMI was a more intensive intervention in terms of time and resources. Since no willingness to pay threshold (WTP) exists for the UPCC and USER-Participation, it is difficult to interpret the cost-effectiveness results; however, it is evident that an ICER (€26,514) for the UPCC is not cost-effective and the ICER (€346) for the USER-Participation has a higher probability of being cost-effective. The probability of ICUR acceptance is 52% using the Dutch willingness to pay threshold (WTP) of €50,000 and therefore we do not consider SMI as a cost-effective alternative in terms of cost/QALYs when compared to EDU.

Sensitivity analyses and subgroup analysis showed robustness of results. Negligible differences in the distribution of bootstrapped ICERs/ICURs on the CE-plane occur when changing the unit price for rehabilitation day treatment and using the friction cost method to calculate productivity costs. Clearer changes in distribution are noticeable when estimating costs from a healthcare perspective, therefore proving the limitation of this perspective in comparison with a societal perspective where all relevant cost categories are being considered. Using a Delta QALY to correct for baseline differences in utility scores and using UK tariffs to calculate QALYs also did not show major differences in comparison with the base cases analyses. The subgroup analysis give no indication for the impact of the imputation method on study findings.

As stated before, the current study was the first to focus on the cost-effectiveness of a stroke-specific self-management intervention. A recent review into the cost-effectiveness of self-management interventions did show the potential of a self-management intervention to be cost-effective for other chronic diseases such as diabetes and low back pain amongst others [8]. It is difficult to compare studies due to major differences in e.g. population, setting and cost measurement. However, the studies were of limited quality and only eight out of twenty two conducted their research from a societal perspective. This shows that the choice of perspective and the limited quality of the methodology may explain differences in cost-effectiveness results.

Previous research into the effectiveness of stroke-specific self-management interventions compared their intervention with care as usual or with waiting lists, instead of a control intervention [40–44]. These studies did not control for the provision of non-self-management components such as peer support and stroke-related information. Therefore, the effectiveness of these self-management interventions might be due to the non-self-management components instead of specific self-management components. This could explain that the current study found the control intervention (EDU) to be effective as well, indicating the importance of choosing a control intervention with regard to cost-effectiveness results. The potential effectiveness of SMI is in line with results from previous research on the effectiveness of stroke-specific self-management interventions [40–44]; however SMI proved not be significantly more effective than EDU.

The potential effectiveness and cost-effectiveness of self-management interventions in chronic diseases is widely recognized, but there is a necessity for tailor-made, disease-specific self-management interventions calling for new evaluations [45]. The underlying mechanism of SMI in the current study was proactive coping. We expected stroke patients to benefit from proactive coping action planning strategies in the current study. However, the stroke-specific SMI and the stroke-specific EDU may have yielded comparable effects because both interventions are group-based educational programs. The essential difference should have the learning of proactive planning strategies, but since no effect was found on the UPCC one may argue that this treatment goal was not reached. Both interventions help people to cope with the consequences of stroke.

Although proactive coping is associated with improved quality of life post-stroke [12], further research should explore other ways of organizing self-management interventions since proactive coping appeared not to be the effective ingredient for SMI in the current study.

### Strengths and limitations

Strengths of the current study include the large sample size and the recruitment of patients at multiple sites increasing the generalizability of study results. Next, there has been no previous economic evaluation research on a stroke-specific self-management intervention; allowing the current study to provide new evidence. Furthermore, the cost-effectiveness analyses were conducted from a societal perspective with a bottom-up method of costing, which we consider to be strengths. In addition, the number of patients who dropped-out was very low. Finally, patients, outcome assessors and research assistants were blinded for the treatment conditions.

The current study was subject to several limitations. First, over 10 % missing can be considered as a drawback of the study. However, we would classify these missings as “completely at random (MCAR)”. Because no specific patron of missing’s could be identified and hence, no systemic differences between baseline characteristics of completers and non-completers were found. Secondly, the imputation of missing data is a sensitive process and subject to assumption. However, previous research recommends individual mean imputation as suitable method for handling missing data [30]. In addition, we investigated the impact of the imputation method and could not found any relevant differences in baseline characteristics of completers and non-completers. In addition, the study outcomes and conclusions in the completers group were comparable to the base case and sensitivity analyses. Second, we found baseline differences on gender and three stroke characteristics (number of months post stroke, type of stroke and stroke history). We did not correct for these baseline differences as it was expected that these would not influence our study findings. We used regression to correct utility baseline differences. Since no preferred method exists, this might be considered a limitation, but we compared this method with a Delta QALY in a sensitivity analysis confirming our decision to choose the regression method [31]. Third, we estimated the productivity costs of partners with a mean hourly wage and a mean age, since limited information was available. Fourth, although we think a follow up period of 12 months is long enough to adequately evaluate the cost-effectiveness of this SMI intervention, it is obviously shorter than the lifelong follow up recommended by the current Dutch guidelines for economic evaluations in health care [46]. A follow up study evaluating self-management intervention in an economic model could fill up this knowledge gap. Fifth, we did not take into account any hospital of centre effects due to the small amount of participating centres. This is considered to be a limitation of this study. Finally, although it is possible to calculate sample sizes for economic evaluations, we performed our sample size calculations on the primary clinical outcomes of the trial. The rather small sample size used in our study (*n* = 113) could therefore theoretically result in not finding relevant differences between the two treatment groups (type 2 errors).

## Conclusions

The current study showed that a stroke-specific self-management intervention based on proactive coping action planning was not a cost-effective alternative in comparison with a stroke-specific education-based intervention for two outcomes measures (UPCC and QALY). The intervention yields more effects and more costs, but the investment needed to reach an acceptable probability that SMI will be cost-effective is undesirable. However, the other outcome measure, on participation (USER-Participation), did show a probability that the self-management intervention could be cost-effective. Based on the current results, implementing SMI for stroke patients is debatable. Discussion must focus on an acceptable willingness to pay threshold for each outcome measure, but based on these study results we do not recommend implementing the self-management intervention in a stroke population. We do recommend exploring the potential cost-effectiveness of self-management interventions in stroke care further using for instance different control interventions, because it has proven to be cost-effective for other diseases. The focus should be also on a different underlying mechanism than proactive coping. Also, despite not being part of this research, it would be very interesting to investigate willingness to pay thresholds for other outcome measure, such as the USER-Participation and UPCC, besides QALYs.

## Data Availability

The datasets used and/or analysed during the current study are available from the corresponding author on reasonable request.

## Abbreviations

SMI: Self-management intervention
EDU: Education-based intervention
RCT: Randomized controlled trial
USER-Participation: the Utrecht Scale for Evaluation of Rehabilitation-Participation
SAN: the Aphasia Scale of the Dutch Aphasia Foundation
CEA: Cost-effectiveness analysis
UPCC: Utrecht Proactive Coping Scale
SD: Standard deviation
CUA: Cost-utility analysis
HRQol: Health-related quality of life
QALY: Quality adjusted life year
GP: General practitioner
SPSS: Statistical Package for the Social Sciences
ICER: Incremental cost-effectiveness ratio
ICUR: Incremental cost-utility ratio
CEAC: Cost-effectiveness acceptability curve
WTP: Willing to pay
NE: Northeast
SE: Southeast
UK: United Kingdom
